# YBX1: A Multifunctional Protein in Senescence and Immune Regulation

**DOI:** 10.3390/cimb46120841

**Published:** 2024-12-13

**Authors:** Wenze Zhang, Ying Liu, Zhe Zhao, Yizhi Zhang, Yujuan Liang, Wanxia Wang

**Affiliations:** 1 The First College of Clinical Medicine, Lanzhou University, Lanzhou 730000, China; aspiring0916@163.com; 2 The First Clinical Medical College, Gansu University of Traditional Chinese Medicine, Lanzhou 730000, China; l15101216275@163.com (Y.L.); zhezhaohappy@163.com (Z.Z.); zhangyizhi1201@163.com (Y.Z.); 18993260748@163.com (Y.L.); 3 NHC Key Laboratory of Diagnosis and Therapy of Gastrointestinal Tumor, Lanzhou 730000, China

**Keywords:** YBX1, cellular senescence, tumor immunity, immunotherapy

## Abstract

The Y-box binding protein 1 (YBX1) is a multifunctional protein with a wide range of roles in cell biology. It plays a crucial role in immune modulation, senescence, and disease progression. This review presents a comprehensive analysis of the specific functions and mechanisms of YBX1 in these areas. Initially, YBX1 is shown to be closely associated with cellular senescence and impacts significant biological processes, including cell proliferation, damage repair, and metabolism. This suggests potential applications in the prevention and treatment of senescence-related diseases. Additionally, YBX1 regulates the immune response by controlling the function of immune cells and the expression of immune molecules. It is essential in maintaining immune system homeostasis and impacts the pathological process of various diseases, including tumors. Lastly, the diverse functions of the YBX1 protein make it a promising candidate for the development of innovative therapeutic strategies for diseases. Comprehensive research on its mechanisms could provide novel insights and approaches for the prevention, diagnosis, and treatment of related diseases.

## 1. Introduction

Y-box binding protein 1 (YBX1) is a multifunctional member of the cold shock domain protein family and serves as a significant transcription factor [1]. It regulates transcription by binding to Y-box sequences found in the enhancer and promoter regions of various genes, thereby modulating the expression of numerous genes associated with cellular proliferation, senescence, survival, drug resistance, and chromosomal instability. This regulation implicates YBX1 in a broad spectrum of biological processes [2]. Additionally, YBX1 is a key member of the RNA-binding protein (RBP) family, playing an important role in regulating RNA modifications and metabolism.

Given its multifunctionality, YBX1 is an indispensable player in cellular biological processes. Its importance is underscored by its role in maintaining genomic stability, facilitating adaptive responses to cellular stress, and contributing to post-transcriptional gene regulation, which collectively influences cellular fate decisions. Notably, aberrant expression or dysregulation of YBX1 has been closely linked to several pathological conditions, particularly cancer, where it is associated with tumor progression, metastasis, and resistance to therapy [3]. Beyond cancer, YBX1 also plays a critical role in autoimmune diseases, senescence-related disorders, and inflammatory processes [4].

Despite the growing body of research on YBX1, most existing reviews have primarily focused on its roles in cancer biology. However, while there is emerging evidence linking YBX1 to immunity and cellular senescence, comprehensive reviews on its roles in these processes are still limited. This review aims to focus on the roles of YBX1 in immunity and senescence, as well as its potential clinical significance.

Unlike existing reviews, this analysis emphasizes YBX1’s role in immunity and senescence, as well as its implications for therapeutic innovation. By exploring the molecular mechanisms and biological impacts of YBX1, this analysis seeks to provide new perspectives and strategies for the treatment and prevention of related diseases, thus contributing to the advancement of medical research and therapeutic innovation.

## 2. The YBX Family: Structure and Function

The YBX (Y-box binding protein) family consists of a group of evolutionarily conserved proteins that share a common structural feature, a conserved cold shock domain (CSD), which enables them to bind both DNA and RNA, playing roles in gene expression regulation [5]. The YBX family is divided into several members, with the most studied being YBX1, along with other members like YBX2 and YBX3. Among the three members of the family, the protein sequences of CSDs share more than 90% identity, especially between YB-1 and YB-3, which were highly conserved, and their CTDs are close in amino acid composition (Arg, Pro, Glu, Gln, and Gly amount to approximately 60% of all residues) and contain a large number of charged residues. In contrast, the N-terminal domains of the three YB proteins are the least homologous, although all are rich in alanine and proline residues [5].

The expression of YBX proteins can be divided into somatic and germ cell-specific expression. In somatic cells, there are only two YBX proteins, YBX1 and YBX3 [5,6]. The mRNA sets bound to YBX1 and YBX3 are almost the same, and they amount to about 80% of the transcriptome [7]. Yet, the specificity of these proteins is somewhat different.

YBX1 is believed to play an important role in basic cellular functions, especially during the early stages of ontogenesis [8]. YBX1’s ubiquitous expression and prominent role in oncogenesis and stress responses make it a critical focus in cancer biology, while YBX3’s more specialized functions in neuronal and muscle tissues underscore its importance in tissue-specific regulatory mechanisms. YBX3 may be implicated in neurodegenerative disorders and muscle-related diseases [6].

Germ-cell-specific YBX2 is expressed in oocytes and testicular germ cells in the stage of spermatogonia to spermatocyte, also observed in placental trophoblasts, as well as in vascular smooth muscle cells in the pulmonary artery, myocardium, and skeletal muscle. unlike cold shock domains of YBX1 and YBX3, the YBX2 CSD has Phe 99 instead of Tyr99. The same preference for Phe is demonstrated by the YBX2 C-terminal domain. This may well affect the RNA-binding properties of YBX2 [9]. The studies of I YBX2s RNA targets were restricted to germ cells, which shows specificity. The literature offers no data on the entire range of YBX2-interacting RNAs, although YBX2 and YBX3 are known to modulate the translation of many mRNAs of the testis, which suggests a wide range of targets [10]. The elevated affinity of YBX2 and YBX3 for mRNAs containing YRS (Y-box protein recognition sequence) is indirectly supported by the fact that the translation inhibition depends on the YRS presence in conditions of a limited YB protein amount [10].

## 3. Overview of YBX1

### 3.1. The Research History of YBX1

In the 1970s, YBX1 was first identified as a major component of messenger ribonucleoprotein particles (mRNPs) in both avian and mammalian cells [11,12], where it was initially referred to as p50. Significant research on this protein began in the 1980s. In 1988, it was discovered that p50 could bind to the Y-box sequence (5′-CTGATTGGGCTCTAA-3′) in the promoter region of MHC class II genes, leading to its designation as Y-box binding protein 1 [13]. Additionally, that same year, YBX1 was identified as a transcription factor regulating the EGFR gene [14], which is associated with cell proliferation, highlighting YBX1’s role in transcriptional regulation through DNA binding. In 1996, it was discovered that YBX1 influences DNA repair processes, thereby protecting cells from damage caused by DNA cross-linking agents [15].

Although YBX1’s interaction with RNA was identified before its interaction with DNA, its comprehensive regulatory roles in RNA were elucidated later. In 1998, YBX1 was found to affect mRNA translation [16]. By 2001, it was established that YBX1 could influence mRNA stability and mediate pre-mRNA splicing [17,18], highlighting its involvement in the post-transcriptional regulation of gene expression.

Since the late 1990s, the association between the YBX1 protein and various diseases, senescence, and immune regulation has been increasingly recognized. In 1990, YBX1 was found to be involved in the clonal expansion and activation of lymphocytes [19]. In 1997, YBX1 was implicated in the progression of breast cancer [20], and in 1998, its involvement in osteosarcoma was reported [21]. In 2005, YBX1 was identified as a stress response molecule that can prevent premature senescence in mouse embryonic fibroblasts [22]. As research into YBX1’s functions progressed, it emerged as a promising therapeutic target. Around 2013, formulations targeting YBX1 were developed and tested for cancer treatment [23].

**Figure 1:**
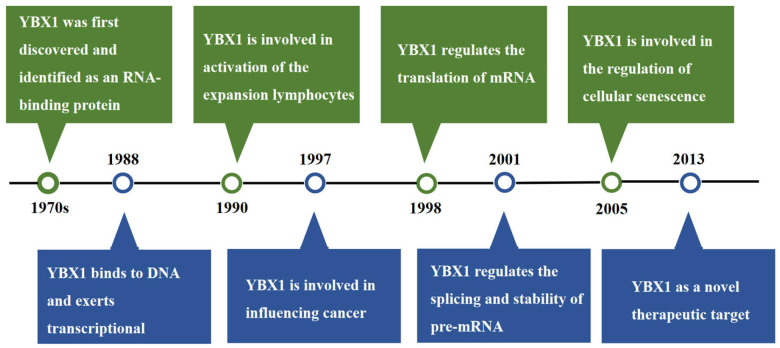
The Research History of YBX1.

As a multifunctional protein, YBX1 regulates gene expression at various levels and is involved in disease progression. Its potential in disease diagnosis and therapy continues to be explored (Figure 1).

### 3.2. Structure and Function of YBX1

The YBX1 gene is situated on the positive strand of chromosome 1, covering 21,388 base pairs within the region from 43,148,089 to 43,169,476. It encodes the YBX1 protein, which consists of 324 amino acids and has a molecular weight of 35,924 Da.

**Figure 2:**
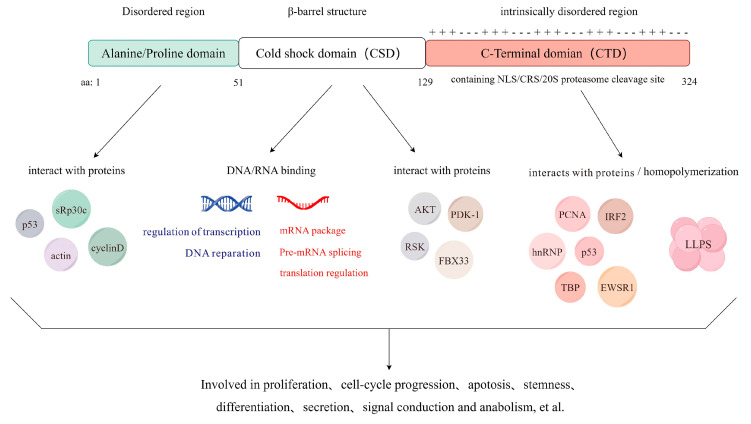
Structure of YBX1. YBX1 is comprised of three primary structural domains: the Alanine/Proline domain (A/P domain), the Cold Shock Domain (CSD), and the C-terminal Domain (CTD). Each of these domains harbors protein-binding sites, facilitating diverse protein-protein interactions that are crucial for YBX1’s multifunctional roles within the cell. Additionally, the CSD uniquely contains nucleic acid-binding sites, enabling YBX1 to engage directly with DNA and RNA. Collectively, these domains mediate complex interactions between YBX1 and both nucleic acids and other proteins, illustrating the protein’s central role in cellular regulatory mechanisms. Created using Figdraw.

YBX1 is structurally composed of three distinct domains: a highly conserved Cold Shock Domain (CSD), an N-terminal domain rich in alanine and proline (A/P domain), and a large C-terminal domain (CTD) characterized by alternating positive and negatively charged amino acids [24]. This unique structural composition enables YBX1 to perform various biological functions, interact with nucleic acids, and form heterodimer complexes with other proteins (Figure 2).

YBX1’s functional diversity arises from its capacity to interact with a broad range of DNA and RNA molecules, implicating its involvement in diverse cellular processes [8]. YBX1 demonstrates an ability to bind to a wide array of DNA and RNA molecules. A DNA binding domain consisting of positively charged aromatic residues on the surface of the β-barrel-like structure in the CSD structural domain of YBX1 can mediate the specific binding of YBX1 to DNA. Initially identified as a transcription factor, YBX1 plays a crucial role in regulating gene expression by specifically binding to DNA sequences in the promoter and enhancer regions of many genes [13,25,26].

The CSD structural domain of YBX1 mediates interaction with RNA through π–π stacking interactions involving four highly conserved aromatic residues: His-87, Phe-85, Phe-74, and Trp-65 [27]. Additionally, The PDB contains several key structural entries related to YBX1 that provide valuable information on its functional mechanisms. For example, PDB entries such as 5YTS and 5YTT describe the crystal structures of the YBX1 cold-shock domain in complex with RNA sequences, which help visualize the specific interactions between YBX1 and RNA molecules, further elucidating its role as an RNA-binding protein. YBX1 functions as an RNA-binding partner and participates in processes such as splicing, translation, mRNA stability, and RNA sorting. These processes are frequently associated with RNA processing within exosomes.

As a crucial m5C reader protein, YBX1 can bind to m5C sites on mRNA through two tryptophan residues (Trp45 and Trp65) in its CSD domain. It specifically recognizes mRNA transcripts modified by 5-methylcytosine (m5C) and promotes mRNA stability in an m5C-dependent manner by recruiting the mRNA stabilizer ELAV-like RNA Binding Protein 1 (ELAVL1) [28]. Additionally, PDB entries like 6KUG and 6A6J provide further details of YBX1’s cold-shock domain in complex with m5C RNA, shedding light on its capacity to recognize and bind modified RNA sequences, a modification crucial for mRNA stability and regulation.

Moreover, YBX1 can bind to mRNA and mediate the formation of “bead-like” mRNP structures through the positively charged amino acid residues in its CTD, thereby inhibiting translation [29,30]. However, when YBX1 undergoes S165 phosphorylation, the CTD bridges to the adjacent CSD, neutralizing the RNA phosphates exposed on its arginine residues and forming an electrostatic zipper. This transformation results in YBX1 forming linear nucleoprotein filaments with mRNA, thus promoting translation [31]. Therefore, the precise spatiotemporal regulation of mRNP translational activity by YBX1 can be achieved through post-translational modifications of the CTD and CSD [32], as well as interactions with protein partners [33].

The A/P and CTD domains are particularly notable for their lack of regular secondary structure, resulting in disordered configurations.

All three structural domains of YBX1 function as multifunctional protein interaction sites and regulatory centers. The CSD domain consists of five-stranded β-barrel-like tertiary structures with conserved sequences, Ribonucleoprotein consensus sequence 1 (RNP1) and Ribonucleoprotein consensus sequence 1 (RNP2) [34], which facilitate both specific and non-specific interactions with nucleic acids. Additionally, the CSD includes various enzyme binding sites, enabling interactions with proteins such as p90 Ribosomal S6 Kinase (RSK), AKT kinase [35], and E3 ubiquitin ligase. The A/P domain contains protein binding sites that enable interactions with various proteins, including the splicing factor SRp30c [36], actin [37], Cyclin D1 [38], and p53. The CTD contains several critical functional sites, including a nuclear localization signal (NLS), a cytoplasmic retention site (CRS), and a cleavage site for the 20S proteasome [34,39]. The type II polyproline helices within the CTD facilitate interactions with key regulatory proteins, including heterogeneous nuclear ribonucleoproteins (hnRNP) [40], transcription factor p53 [41], E3 ubiquitin ligase Retinoblastoma Binding Protein 6 (RBBP6) [42], and Iron Regulatory Protein 2 (IRP2) [43]. Furthermore, the alternating arrangement of basic and acidic amino acids within the CTD’s disordered structure contributes to YBX1’s homopolymerization [44,45], a process known as phase separation.

All three domains of YBX1 can mediate its interactions with proteins. According to the STRING database (https://www.string-db.org, accessed on 8 December 2024), YBX1 primarily interacts with key proteins involved in RNA metabolism and stability. These include Heterogeneous Nuclear Ribonucleoprotein D (HNRNPD) and Fragile X Mental Retardation 1 (FMR1), which participate in RNA transport; Poly(A) Binding Protein Cytoplasmic 1 (PABPC1), ELAVL1, and Cold Shock Domain Containing E1 (CSDE1), which are involved in mRNA stability; Heterogeneous Nuclear Ribonucleoprotein A2/B1 (HNRNPA2B1), Heterogeneous Nuclear Ribonucleoprotein K (HNRNPK), and Insulin-like Growth Factor 2 MRNA Binding Protein 1 (IGF2BP1), which are associated with RNA metabolism and translation regulation; as well as Heat Shock Protein Family A (Hsp70) Member 1A (HSPA1A) and Heat Shock Protein Family A (Hsp70) Member 1B (HSPA1B), which are involved in protein folding. These interactions suggest that YBX1 plays a significant role in RNA metabolism, stability, and translation regulation through its interactions with various proteins.

YBX1 can form reversible amyloidogenic fibrils under high ionic strength conditions. These fibrils exhibit amyloid-like properties, such as Congo red staining and characteristic X-ray diffraction patterns, but unlike typical amyloid fibrils, they are reversible and can disassemble under nearly physiological conditions (e.g., 0.15 M KCl). The CSD domain plays a crucial role in this process, while the A/P domain and CTD contribute to the formation of shadow protofibrils [46]. These protofibrils are intermediate, partially formed fibrillar structures that serve as the foundation for the formation of cytoplasmic granules by the YBX1 protein [46]. The distinct structural features of these three domains facilitate YBX1’s interactions with other proteins and nucleic acids. These interactions regulate various cellular processes, such as cell signaling, anabolic and catabolic metabolism, and cell growth, thereby maintaining normal cellular function.

YBX1 is primarily localized in the cytoplasm, but it translocates to the nucleus in response to external stimuli [47], internal stress, activation of signaling pathways [35], and changes in the cell cycle [48]. Once in the nucleus or cytoplasm, YBX1 serves as a new RNA 5-methylcytosine reader. It plays a crucial role in regulating mRNA metabolism at various stages of transcription, splicing, packaging, stabilization, and translation [49]. YBX1 is also involved in DNA damage repair to maintain nucleic acid homeostasis [50]. Furthermore, it may function as an extracellular mitogen that promotes cell migration and proliferation and contributes to the composition of extracellular exosomes that affect intercellular signaling communication [51].

YBX1 is a widely expressed protein found in a variety of tissues throughout the body. Its expression is particularly high in tissues that are involved in rapid cell proliferation, stress responses, and apoptosis regulation.

YBX1 is expressed in a broad range of tissues, with its highest expression levels in those undergoing rapid cellular turnover, such as the brain, liver, testes, ovaries, and skin. Its roles in regulating cell proliferation, stress responses, and apoptosis make it crucial for normal development and tissue homeostasis. Additionally, its dysregulation is linked to various diseases, including cancer and neurodegenerative conditions [52].

### 3.3. Regulation of YBX1 Expression and Its Association with Disease

The regulation of YBX1 synthesis involves a multitude of factors, with the E box sequence (CATCTG), GATA motif, and GGAA motif within its gene promoter emerging as pivotal elements governing transcriptional activity. Various transcription factors exhibit the capacity to modulate YBX1 transcription by binding to these regulatory regions. Stimuli such as cell differentiation, epithelial–mesenchymal transition (EMT), and proliferation can incite the activation of YBX1 transcription. For instance, the engagement of the c-Myc-Max complex and the transcription factor Twist with the E box situated in the YBX1 gene promoter serves to amplify YBX1 transcriptional output, thereby fostering an augmentation in its expression levels [53,54]. Such regulatory mechanisms underscore the intricate interplay of molecular components orchestrating the dynamic regulation of YBX1 synthesis in response to diverse cellular cues and environmental stimuli.

At the translational level, various proteins exert influence over YBX1 synthesis by binding to regulatory sequences within the untranslated region of YBX1 mRNA. For instance, the specific interaction between hnRNP Q and the regulatory element within the 3′UTR region of YBX1 mRNA leads to the liberation of the positive regulator of YBX1 mRNA translation, the poly(A) binding protein (PABP). This culminates in the inhibition of YBX1 mRNA translation [55,56]. Moreover, the activation of the mTORC1: Mechanistic Target of Rapamycin Complex 1 (mTORC1) signaling pathway enhances YBX1 translation [57]. Furthermore, YBX1 itself can serve as a negative regulator, impeding its own expression by binding to its mRNA.

At the post-translational level, YBX1 undergoes modifications, including phosphorylation, acetylation, methylation and ubiquitination, in response to various kinases, leading to alterations in its biological activity (Table 1). Phosphorylation is the most widely occurring post-translational modification in YBX1. YBX1 phosphorylation sites contain S102 [35], S176 [58], S165 [59], Y162 [60], S209 [61], Y188 and Y281 [62]. For example, AKT and ERK can phosphorylate the serine residue at position 102 of YBX1 through their downstream target, RSK, promoting its translocation from the cytoplasm to the nucleus [63]. S165 phosphorylation is critical for the activation of NF-kB by YBX1 [59]. PLK1 interacts with YBX1, directly phosphorylates S174 and S176 of YBX1, promotes its nuclear translocation, and thereby inhibits apoptosis and DNA damage in glioblastoma stem cells (GSCs) [58].

YBX1 can also be acetylated in pathological states or in cancers. YBX1 acetylation at K301/304 links to inflammation and vascular damage [64], while K81 acetylation promotes YBX1 nuclear translocation, driving metastasis-related protein activation in cancer [32]. YBX1 also undergoes methylation, primarily at arginine residues such as R199, R200, R239 [65], and R205 [66], catalyzed by enzymes like Protein Arginine Methyltransferase 5 (PRMT5). This modification is crucial for processes like NF-κB activation, gene transcription, and protein interactions.

Moreover, the stability of YBX1 is subject to regulation by protein degradation mechanisms. In conditions of DNA damage stress, apoptosis, or cell proliferation defects, the stability of the YBX1 protein is modulated by degradation pathways such as the ubiquitin-proteasome system, thereby influencing its protein levels and functional longevity within the cell [42,67]. For example, Seven in Absentia Homolog 1 (SIAH1) ubiquitinates YBX1 at K304 to reduce drug resistance of epithelial ovarian cancer cells to cisplatin [68]. Additionally, besides proteasomal degradation, YBX1 may also undergo degradation through autophagic pathways. This intricate regulation of YBX1 protein turnover is vital for maintaining cellular homeostasis and effectively responding to intracellular and extracellular signals.

The aberrant expression of YBX1 is associated with various diseases, including cancer, inflammatory conditions, and neurodegenerative disorders. In cancer, specifically, YBX1 expression is closely linked to the progression of multiple malignancies. For example, circNEIL3 directly interacts with YBX1, promoting Neural Precursor Cell Expressed Developmentally Down-Regulated Protein 4-Like (Nedd4L)-mediated proteasomal degradation of YBX1, thereby reducing its levels and inhibiting colorectal cancer metastasis [69]. Conversely, Immune-related GTPase (IRGM) binds directly to YBX1, facilitating its phosphorylation and nuclear localization, which subsequently upregulates Programmed Death-Ligand 1 (PD-L1) expression and promotes the malignant progression of hepatocellular carcinoma [70].

**Figure 3:**
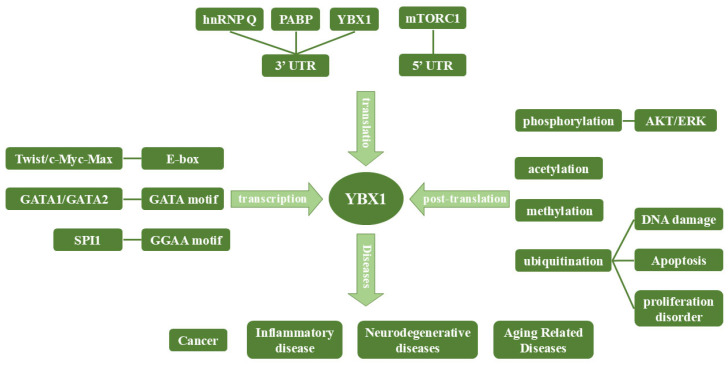
Regulation of YBX1 Expression. YBX1 expression is regulated at multiple levels, including transcription, translation, and post-translational modification. Key regulatory mechanisms involve interactions with transcription factors and untranslated regions. Post-translational modifications, such as phosphorylation and ubiquitination, further influence its activity. Dysregulation of YBX1 contributes to the development of cancer, inflammatory diseases, neurodegenerative diseases, and aging-related disorders. hnRNP Q: Heterogeneous Nuclear Ribonucleoprotein Q; PABP: Poly(A)-Binding Protein; mTORC1: Mechanistic Target of Rapamycin Complex 1; UTR: Untranslated Region; ERK: Extracellular Signal-Regulated Kinase.

In conclusion, YBX1 is regulated by multi-level, multi-step, and multi-factorial mechanisms. These regulatory processes maintain a dynamic balance within the cell, ensuring appropriate YBX1 expression and normal biological activity, which are essential for proper cellular functions (Figure 3).

**Table 1:** YBX1’s post-translational modifications.

Site on YBX1	Location in YBX1 Structure	Type of PTM	References
S102	CSD	Phosphorylation	[35]
S176	CSD	Phosphorylation	[58]
S165	CSD	Phosphorylation	[59]
Y162	CTD	Phosphorylation	[60]
S209	CSD	Phosphorylation	[61]
Y188, Y281	CTD	Phosphorylation	[62]
K301/304	A/P Domain (N-terminal region)	Acetylation	[64]
K81	A/P Domain (N-terminal region)	Acetylation	[32]
R199, R200, R239, R205	CSD/CTD linker region	Methylation	[65,66]
K304	A/P Domain (N-terminal region)	Ubiquitination	[68]

PTM: Post-Translational Modification; A/P domain: Alanine/Proline domain; CSD: Cold Shock Domain; CTD: C-Terminal Domain.

## 4. YBX1 Regulates Cellular Senescence and Impacts the Progression of Tumors and Age-Related Diseases

Cellular senescence is defined as a state in which diploid cells are in a state of stable cell cycle arrest accompanied by an enhanced secretory phenotype and resistance to cell death, resulting in impaired cell proliferation [71], typically due to DNA damage. While it is crucial for regular tissue development, tissue repair, immune cell attraction, and the prevention of tumor growth [72], it may also result in persistent inflammation and illnesses if senescent cells persist in producing senescence-associated secreted phenotypic (SASP) factors, which have been known to contribute to the development of cancer [73].

The regulation of cellular senescence is a complex process that involves various factors and hierarchies to ensure the organism’s internal environment is maintained. Primarily acting as an anti-aging gene [74], YBX1 exerts significant influence during the process of cellular senescence. It modulates cellular senescence by regulating cell cycle dynamics [75], DNA damage response (DDR) [76], and SASP [71]. Through these mechanisms, YBX1 contributes to the development and progression of cellular senescence, underscoring its critical function in the maintenance of cellular integrity and longevity.

### 4.1. YBX1 Impedes Cellular Senescence by Modulating Cell Cycles

YBX1 orchestrates the transcription and translation of cell cycle-related proteins and interacts with regulatory proteins governing the cell cycle. By finely regulating these processes, YBX1 assumes a pivotal role in impeding cellular senescence. Research reveals that YBX1 can bind to the promoter region of the Cyclin-Dependent Kinase (CDK) inhibitor p16INK4A, thereby repressing its transcription [75,77]. Additionally, YBX1 binds to the 5′UTR region of p16INK4A mRNA, bolstering its translation [78]; albeit, under normal circumstances, YBX1 primarily governs p16INK4A expression at the transcriptional level. Moreover, YBX1 governs the expression levels of CCN Domain Binding Protein 1 (CCNDBP1) [79], fostering the assembly of the Cyclin D-CDK4/6 complex.

YBX1’s release from senescence-associated heterogeneous ribosomes curtails mRNA binding to genes associated with senescence [80]. Furthermore, YBX1 exerts direct control over the transcription of Growth Arrest and DNA Damage-Inducible 45 alpha (Gadd45a) and Glycogen Synthase Kinase 3 beta (GSK3B), thereby dampening CyclinE1 expression [81,82], which in turn perturbs normal DNA replication and progression through various cell cycle phases. Further underscoring its regulatory role, YBX1 enhances the translational efficiency of Aurora Kinase A (AURKA) mRNA, augmenting protein levels and fostering the formation of the Cyclin B/CDK1 complex, thereby facilitating the transition from G2 to M phase [83]. Lastly, YBX1’s interaction with the p53 protein inhibits its activity, diminishing levels of the downstream target gene p21CIP1 and promoting the formation of the Cyclin E-CDK2 complex, thereby enabling cells to transition from the G1 to S phase [22,84]. In essence, YBX1 adeptly modulates cyclin/CDK complex formation and activity through diverse mechanisms, thus ensuring proper cell cycle progression and staving off cellular senescence.

### 4.2. YBX1 Suppresses Cellular Senescence by Both Preventing and Repairing DNA Damage Response

DDR is widely recognized as a primary trigger of cellular senescence [73], with YBX1 playing a crucial role in modulating its initiation and progression. Reduced YBX1 levels can lead to nucleic acid damage. As a cellular stress response factor, YBX1 plays a critical role in mitigating oxidative stress by reducing the accumulation of reactive oxygen species (ROS), thereby lowering the incidence of DDR [76]. Research indicates that defects in YBX1 can exacerbate the effects of ionizing radiation on medulloblastoma (MB) cells, resulting in MB cell senescence [85]. YBX1 also actively participates in the repair of nucleic acid damage. It functions as a critical splicing factor, preventing the improper splicing of genes that can cause nucleic acid damage during the senescence of bone marrow mesenchymal stem cells (BMSCs). Studies have demonstrated that YBX1 can bind to the Breast Cancer 1 (BRCA1) promoter region to promote its transcription, form a complex with U2 Small Nuclear RNA Auxiliary Factor 65 kDa (U2AF65) and bind to RAD51 Recombinase (RAD51) mRNA to enhance RAD51 expression, and assemble protein complexes at damaged DNA double-strand breaks to facilitate homologous recombination repair (HRR) of broken strands [86,87]. Additionally, YBX1 can undergo poly-ADP-ribosylation (PAR) modification, particularly at its C-terminal region (amino acids 219–324), which is rich in arginine residues, during its interaction with damaged DNA [88]. This modification plays a role in regulating DNA repair, as poly-ADP-ribosylation is known to affect the binding of YBX1 to DNA and its subsequent involvement in the DNA damage response [88]. In summary, YBX1 regulates DDR occurrence and repair in multiple ways, thus mitigating the onset of cellular senescence.

### 4.3. Phosphorylated YBX1 Promotes Cellular Senescence by Regulating SASP

SASP is a hallmark of senescent cells and mediates a range of pathophysiological responses. YBX1 can impact the production and release of SASP factors, thus affecting the onset of cellular senescence. Key SASP factors include the pro-inflammatory cytokines interleukin 6 (IL-6), interleukin 8 (IL-8), and interleukin 1α (IL-1α) [71]. The phosphorylated YBX1 protein is capable of promoting the translation of IL-1α mRNA by binding to its 3′UTR region, thereby activating the transcription of critical SASP factors like IL-6, IL-8, and C-X-C Motif Chemokine Ligand 1 (CXCL1) [89]. Additionally, YBX1 can encapsulate SATII RNA in the extracellular vesicles (sEVs) of senescent cells and act as a SASP factor in recipient cells, leading to senescence and promoting the expression of inflammatory SASP genes in such cells [90]. In summary, YBX1 plays a pivotal role in cellular senescence through its regulation of SASP factor expression and secretion, as well as its influence on the autocrine and paracrine secretion of SASP factors.

### 4.4. YBX1 Regulates Senescence-Related Diseases

The aberrant expression of YBX1 is closely associated with tumorigenesis and age-related diseases such as osteoporosis. In age-related osteoporosis, the expression of YBX1 in BMSCs decreases with aging. By regulating the splicing of transcripts such as Fibronectin 1 (Fn1), which plays a role in cell adhesion and migration; Neuropilin 2 (Nrp2), involved in angiogenesis and neuronal development; Sirtuin 2 (Sirt2), a deacetylase associated with cellular stress responses and the regulation of senescence; Osterix (Sp7), a key transcription factor for osteoblast differentiation; and Secreted Phosphoprotein 1 (Spp1), which regulates bone mineralization and bone metabolism, YBX1 affects the osteogenic differentiation and senescence of BMSCs, ultimately mediating the progression of osteoporosis [91]. In hepatocellular carcinoma (HCC), YBX1 modulates the expression of nucleotide metabolic enzymes Ribonucleotide Reductase M2 (RRM2), Thymidine Kinase 1 (TK1), and Thymidylate Synthase (TYMS), leading to increased nucleotide metabolism and DNA synthesis, which are essential for cancer cell proliferation [92]. Additionally, YBX1 regulates cyclinD1 [93], a key cell cycle regulator, collectively influencing the normal progression of the HCC cell cycle and mediating the occurrence of HCC senescence, thereby suppressing the malignant progression of tumors.

**Figure 4:**
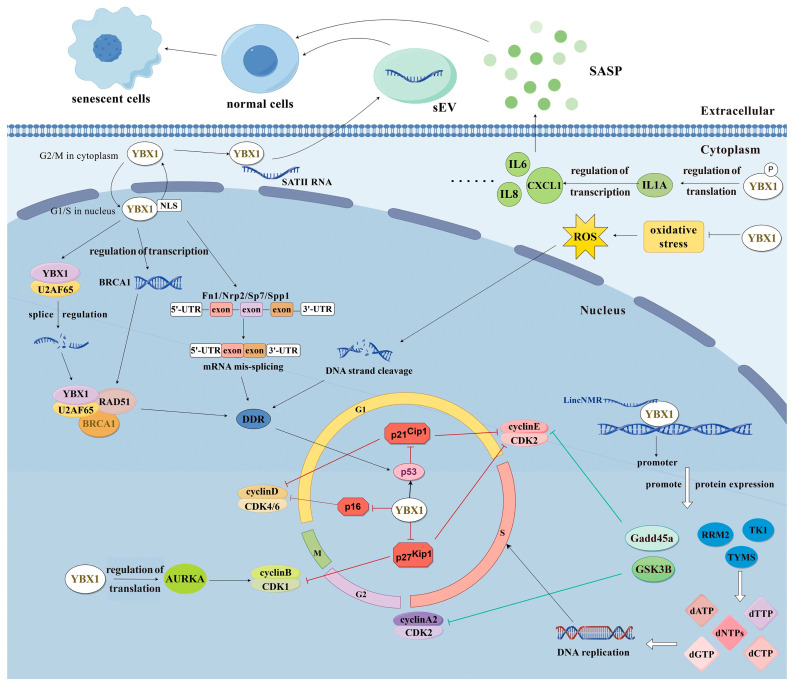
YBX1 is involved in the development of cellular senescence. YBX1 regulates cellular senescence through multiple pathways. It influences the progression of the cell cycle by modulating the levels of cell cycle-associated proteins. Additionally, YBX1 participates in the regulation of the DDR by affecting both the accumulation of DNA damage and its repair mechanisms. Moreover, YBX1 impacts the Senescence-Associated Secretory Phenotype (SASP) by influencing the production and secretion of SASP factors. YBX1 also modulates the levels of ROS and sEV, both of which play critical roles in cellular senescence and intercellular communication. These pathways collectively contribute to YBX1’s role in controlling cellular senescence, integrating various regulatory mechanisms to maintain cellular integrity and function. Created using Figdraw.

YBX1’s role in regulating cellular senescence and senescence-related diseases has become increasingly significant. It participates in several pathways, including cell cycle regulation, DDR, and the expression and secretion of SASP factors (Figure 4). By delving deeper into YBX1’s involvement in cellular senescence, we can gain a better understanding of the molecular regulatory mechanisms that govern it. This knowledge can then be used to develop interventions for senescence-related diseases, which would help with diagnosis and treatment.

## 5. YBX1’s Dual Role in Immune Regulation: Implications for Autoimmune Diseases and Tumor Immunity

YBX1 is an essential component of the immune response regulatory network, with a significant influence on the regulation of inflammation, immune cell function, and the expression of antigens. YBX1 is intricately linked to proteins involved in immune cell interactions and signaling. Furthermore, YBX1 can impact immune response regulation through various mechanisms and pathways.

### 5.1. YBX1 Regulates Immune Molecules Involved in the Development of Autoimmune Diseases

YBX1 is crucial in regulating the immune system and preventing autoimmune diseases. It can control the expression of various immune and inflammatory factors, influence immune cell activation, differentiation, and infiltration, and manage the onset and progression of inflammatory and autoimmune diseases.

#### 5.1.1. YBX1 Regulates Immune Status by Influencing the Expression of Immune Molecules or Binding to TNFR

YBX1 primarily regulates immune responses by modulating the expression of immune molecules. It enhances the expression of IL-6, a pro-inflammatory cytokine involved in immune response, inflammation, and the regulation of acute phase reactions, and Interleukin-2 (IL-2), a key cytokine for T-cell proliferation, immune activation, and the maintenance of immune tolerance, by stabilizing their mRNAs through binding to their UTR regions [94,95,96]. Additionally, YBX1 interacts with Tumor Necrosis Factor Receptor (TNFR) and Tumor Necrosis Factor Alpha (TNFα), influencing the transcription of TNFα, a cytokine involved in inflammation and immune regulation [97]. YBX1 also promotes the expression and nuclear translocation of NF-κB2, thereby upregulating the NF-κB signaling pathway [98]. This activation increases the expression of the C-X-C motif chemokine receptor 1 (Cxcr1) gene and modulates the immune response by binding to chemokines. Conversely, YBX1 acts as an active component of the mRNA attenuation complex, facilitating the degradation of various immune response-related cytokine transcripts and regulating the expression of CXCL1, a chemokine that plays a crucial role in neutrophil recruitment and the promotion of inflammatory responses during immune reactions [99]. Research also indicates that phosphorylated YBX1 can enhance the expression of Chemokine Ligand 5 (CCL5), a cytokine involved in recruiting immune cells to sites of inflammation, and PD-L1, which plays a key role in tumor immune evasion by inhibiting T-cell activity, by binding to their promoter regions [100,101,102]. This may contribute to the promotion of tumor immune evasion.

#### 5.1.2. YBX1 Exerts Multiple Important Roles in the Pathogenesis of Autoimmune Diseases

YBX1 plays a pivotal role in autoimmune disease development by modulating immune cell activation and the expression of immune molecules. Guanidinylation of YBX1 activates the Notch-3 signaling pathway [103], which is crucial for regulating T-cell differentiation and orchestrating the immune response. Moreover, YBX1 enhances the expression of Interleukin-10 (IL-10), an anti-inflammatory cytokine that plays a key role in regulating immune responses and inhibiting excessive inflammation, and Transforming Growth Factor Beta (TGFβ), a cytokine involved in immune regulation, fibrosis, and tissue repair, by stabilizing their mRNAs, thereby impacting the progression of systemic lupus erythematosus (SLE) and type I diabetes, both of which are immune-mediated diseases [103,104]. Additionally, YBX1 nuclear translocation can exacerbate other autoimmune diseases, like SLE, by upregulating Multidrug Resistance Protein 1 (MDR1) transcription and increasing membrane P-glycoprotein (p-gp) expression [105]. The transcriptional regulation of MDR1 by YBX1 could contribute to therapeutic resistance in SLE, complicating the use of immunosuppressive drugs such as glucocorticoids [106]. Targeting YBX1 could thus enhance treatment efficacy by reducing P-gp levels.

YBX1 acts as an autoantigen in the P-body of serum from primary biliary cirrhosis patients, prompting autoimmune responses and autoantibody production, thus contributing to autoimmune disease development [107]. In SLE, YBX1 serves as an autoantigen, aiding in diagnosis, treatment, and prognosis [108]. Overall, YBX1 significantly influences autoimmune and chronic inflammatory disease development by regulating immune-inflammatory factors, activating signaling pathways, mediating immune cell activation and infiltration, and modulating immune responses.

Although current studies have established YBX1 as a regulator of immune-inflammatory responses, further research is needed to delineate its role in specific autoimmune diseases, particularly through large-scale cohort studies and mechanistic experiments. By targeting the YBX1 signaling axis, it is possible to modulate innate and adaptive immunity, adjust the secretion of immune-inflammatory factors, and prevent autoimmune damage. Given its significant impact on disease pathogenesis and development, YBX1 presents a promising therapeutic target for treating these types of illnesses.

### 5.2. YBX1 Regulates Anti-Tumor Immunity as a Potential Regulatory Target for Tumor Immunotherapy

#### 5.2.1. YBX1 in the Regulation of Tumor Immune Evasion

In the context of cancer prevention and treatment, countering the immune evasion tactics employed by tumor cells represents a formidable challenge. YBX1 emerges as a pivotal regulator in orchestrating this phenomenon, exerting its influence on the infiltration of immune cells and the expression of immune molecules within the tumor microenvironment.

Research indicates that heightened YBX1 expression can promote immune escape in tumor cells. Its interaction with phosphorylated S6 kinase 1 (p-S6K1), facilitated by IRGM, fosters YBX1 phosphorylation and subsequent nuclear translocation [70]. Conversely, direct interaction with FLII can impede its nuclear localization. Within the nucleus, YBX1 binds to the promoter sequences of PD-L1, an immune checkpoint protein, thereby boosting its transcription [109]. Consequently, this triggers PD-L1-mediated apoptosis of CD8+ T cells and upregulates immunosuppressive molecules like IL-10 and TGF-β.

YBX1 also facilitates tumor immune evasion by modulating immune cells. YBX1’s ability to bind to RAN transcripts influences the expression of IL-4 and the infiltration l evels of CD4+ Th2 cells [110]. Additionally, YBX1 promotes the polarization of M1 macrophages towards a tumor-supportive M2 phenotype [111]. Overall, YBX1 fosters an immunosuppressive tumor microenvironment through diverse pathways, culminating in tumor immune evasion and exacerbation of malignancy. This multifaceted role underscores YBX1’s potential as a therapeutic target for strategies aimed at thwarting tumor immune evasion.

#### 5.2.2. YBX1 in Modulating Anti-Tumor Immune Responses and Immunotherapy

YBX1 plays a crucial role in tumor immunotherapy by orchestrating tumor immunity and shaping the anti-tumor immune response through diverse mechanisms. Firstly, YBX1 enhances the effectiveness of immunotherapeutic interventions by modulating the anti-tumor immune response. Its deletion heightens the sensitivity of renal cell carcinoma to interferon therapy and modulates the activity of monocytes, the exhaustion status of T cells, and the phenotypic alterations of macrophages [111]. Additionally, YBX1 influences tumor-associated angiogenesis and invasion [112,113] and augments the lytic activity of oncolytic adenoviruses against YBX1-positive tumor cells, thereby promoting the release of tumor-associated antigens and cytokines [114,115], or acting directly as a tumor-associated antigen to activate both humoral and cellular immunity. This instigates anti-tumor immune responses and eliminates tumor cells. For instance, YBX1, serving as a tumor antigen linked with neuroblastoma and recurrent melanoma, can trigger specific T cell anti-tumor immune responses, which are further potentiated by regulatory T cell depletion [116,117,118]. Moreover, YBX1-dependent lysosomal adenovirus can synergize with CDK4/6 inhibitors to induce a reversal of tumor immunogenicity, resulting in heightened expression of HLA class I molecules, suppression of regulatory T cells, and augmentation of inflammatory anti-tumor responses, thereby enhancing local tumor control [119].

Secondly, the risk prediction models incorporating stemness, methylation, and moonlighting genes associated with YBX1 can furnish valuable insights into the prognosis and survival of tumor patients, serving as pivotal prognostic tools for predicting outcomes and survival rates [120,121,122,123,124].

Lastly, YBX1 can modulate the efflux of therapeutic agents. Its regulatory role in tumor-associated genomes has garnered considerable attention, and hyaluronic acid can induce nuclear translocation of YBX1, promoting transcriptional activation of MDR1 and expression of membrane p-gp, thereby influencing the efficacy of tumor immunotherapy drugs [125].

Elevated endogenous YBX1 levels suppress tumor immunity, and exogenous YBX1-related agents enhance tumor immunity. Thus, YBX1 emerges as a promising target for immunotherapy due to its dual role in the immune system, tumor immune responses, and immunotherapeutic outcomes regulation.

#### 5.2.3. YBX1 in the Construction of Tumor Risk Prediction Models

YBX1’s involvement in constructing tumor risk prediction models is noteworthy.

Extensive research has elucidated YBX1’s multifaceted role as an oncoprotein, demonstrating its interactions with various genes controlling RNA methylation, RNA-binding proteins, tumor-associated macrophages (TAMs), stemness-related genes, immune-related genes (IRGs), and epigenetic-related genes (EPGs). These interactions contribute to the construction of risk prediction models for HCC based on the expression profiles of YBX1 and related genes [121,126,127,128,129,130,131]. Additionally, YBX1 collaborates with m5C regulatory genes to develop risk prediction models for prostate cancer and kidney renal papillary cell carcinoma (KIRP) [132,133] and with genes associated with tumor-infiltrating CD8+ T cells to establish risk prediction models for papillary renal cell carcinoma (papRCC) [134]. These models provide insights into the levels of immune cell infiltration in the tumor microenvironment (TME), the potential for immune evasion, the expression levels of immune checkpoints, and the efficacy of immunotherapy. They also assess the anti-tumor immune response capacity, guiding the selection of tailored treatment approaches effectively. For instance, the risk prediction model for HCC, incorporating stemness-related genes, stratifies patients into high and low-risk groups with distinct profiles of activated CD4+ memory T cells, CD8+ T cells, and M1 macrophages in the TME. This stratification facilitates the prediction of HCC’s anti-tumor immune response potential, enabling personalized clinical treatment strategies [121].

These findings illustrate that YBX1 can effectively predict cancer development and the strength of anti-tumor immune response and underscore the pivotal role of YBX1 in advancing diagnostic and therapeutic tools for cancer management, highlighting its significance in oncology research.

#### 5.2.4. YBX1 in Cancer Therapy

**Figure 5:**
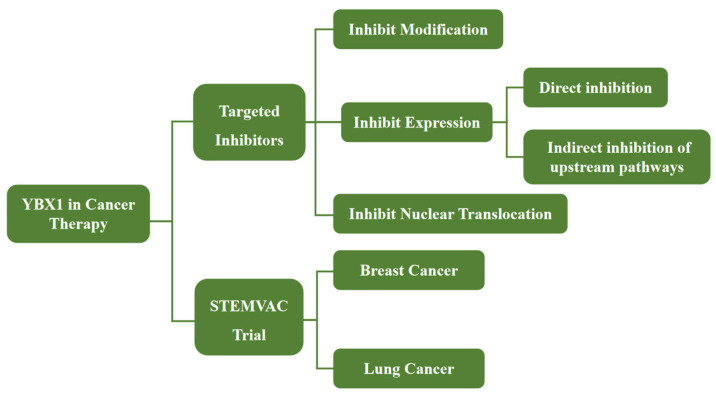
YBX1 in cancer therapy. The role of YBX1 in cancer therapy includes its involvement in targeted inhibitors and the STEMVAC trial. Targeted inhibitors regulate YBX1 by inhibiting its modification, expression, and nuclear translocation, either directly or by affecting upstream pathways. The STEMVAC trial highlights the application of a DNA plasmid vaccine targeting YBX1 and other antigens in breast and lung cancers, showcasing its potential in immunotherapy.

YBX1 is highly expressed in various cancers, and its expression level and cellular localization are closely associated with cancer progression, multidrug resistance, and poor prognosis. Consequently, YBX1 can serve as an effective diagnostic and prognostic biomarker, aiding in tumor diagnosis and predicting invasiveness and recurrence risk. In recent years, targeted therapies against YBX1 and related agents have been developed and tested for cancer treatment (Figure 5).

Current formulations targeting YBX1 primarily influence tumor progression by modulating its expression, modification, and nuclear translocation. Nuclear translocation inhibitors block YBX1’s oncogenic activity in the nucleus by targeting its interactions with nuclear transport-related proteins. For example, 2,4-dihydroxy-5-pyrimidinyl imidothiocarbamate (DPI) inhibits the malignant phenotype of breast cancer by preventing YBX1’s nuclear translocation and the activation of its downstream target genes [135]. YBX1 expression inhibitors directly or indirectly downregulate YBX1 expression by inhibiting the activation of its upstream signaling pathways. For instance, 7-hydroxyisatin enhances the sensitivity of HepG2 cells to actinomycin D therapy by suppressing YBX1 expression [136]. Similarly, BEZ235 downregulates YBX1 expression by inhibiting the PI3K/mTOR pathway, thereby suppressing the malignant proliferation of colorectal cancer cells [137]. YBX1 modification inhibitors reduce YBX1 activity by affecting its acetylation or phosphorylation. For example, MS-275 promotes YBX1 acetylation at K81, enhancing its binding to the 3′UTR region of Nuclear Factor Erythroid 2-Related Factor 2 (NRF2) mRNA, which increases NRF2 translation and decreases ROS in sarcoma cells, ultimately inhibiting sarcoma metastasis [32].

In addition to formulations targeting YBX1, there are clinical trials investigating cancer therapies that focus on YBX1. One notable example is the STEMVAC trial, a DNA plasmid-based vaccine encoding five antigens [Mouse Double Minute 2 Homolog (MDM2), YBX1, SRY-Box Transcription Factor 2 (SOX2), Cell Division Cycle 25B (CDC25B), Cluster of Differentiation 105 (CD105)] associated with breast cancer stem cells [138]. This vaccine effectively activates the body’s anti-tumor immune response to kill tumor cells. YBX1 has been identified as one of the immunogenic proteins targeted in STEMVAC trials for breast and lung cancers, contributing to the development of novel therapies and improving treatment efficacy for these cancers.

The importance of YBX1 in cancer treatment is becoming increasingly recognized. Targeting YBX1 may significantly enhance patient prognosis and quality of life by improving the effectiveness of cancer therapies.

Thus, YBX1 plays a pivotal role in tumor immunity, molecular regulation, auto-immune diseases, and immunotherapy (Table 2). YBX1 can regulate the immune response and immune cell activation by affecting the expression of various immune factors, including interleukins, chemokines, and tumor necrosis factors, participating in anti-tumor immunity. It can also be used to construct risk prediction models to forecast tumor occurrence, progression, and the efficacy of immunotherapy, thereby providing guidance for the prognosis and survival of cancer patients. YBX1 is crucial in tumor immune evasion and progression. Additionally, YBX1 can modulate the progression of autoimmune diseases such as SLE and type 1 diabetes by affecting signal pathway activation and the production of autoantigens and autoantibodies. A deeper investigation into the relationship between YBX1 and immunity will enhance our understanding of the specific molecular mechanisms of immune modulation and the potential applications of YBX1 in disease diagnostics and therapeutics. This knowledge could significantly support the development of novel immunotherapeutic strategies, contributing to the advancement of precision and personalized treatment approaches. Consequently, it is imperative that we continue to focus on elucidating the role of YBX1 in immune regulation to further the progress of life sciences.

In summary, YBX1 plays a critical role in inhibiting cellular senescence and modulating tumor immune evasion. Its involvement in regulating tumor immunity is well-established, and targeting the YBX1 signaling axis holds the potential for reversing tumor immune escape and multidrug resistance, activating the immune response, and restoring the antitumor effects of chemotherapy. This dual action can significantly enhance the antitumor response, making YBX1-targeted immunotherapy combination therapy a promising approach with broad application potential. These advancements are expected to provide a crucial theoretical foundation for the development of innovative immunotherapy strategies.

## 6. Conclusions

YBX1 is a multifunctional protein involved in a variety of cellular functions, and its aberrant expression is closely linked to numerous diseases, including cancer, immune disorders, and aging-related conditions. During disease development, YBX1 interacts with mRNA in the cytoplasm as part of mRNPs, impacting mRNA stability and translational regulation. Within the nucleus, YBX1 can act as both a suppressor and activator, coordinating the transcription of multiple genes that influence biological processes such as aging and immune responses.

This review begins by exploring the fundamental structure and functions of YBX1, with a focus on its roles and mechanisms in cellular senescence and immune response modulation. YBX1’s functional diversity is highlighted, summarizing recent advancements in research on cellular senescence and immune regulation. In cellular senescence, YBX1 primarily influences the regulation of aging by affecting cell cycle-associated protein inhibitors, DNA damage-associated proteins, and the SASP. In immune responses, YBX1 mainly impacts the activation and infiltration of immune cells, expression of immune molecules, antigen presentation, and immune-related signaling pathways, playing a regulatory role in autoimmune diseases, tumor immunity, and immunotherapy. Through its regulatory effects on cellular senescence and immune responses, YBX1 contributes to the onset and progression of cancer and immune diseases, affecting their pathological characteristics.

This review primarily summarizes the impact of YBX1 on immune aspects, noting a scarcity of studies on YBX1’s role in regulating immune responses through cellular senescence. Research indicates that SASP factors secreted by senescent cells can not only promote the senescence of neighboring tumor cells through paracrine effects but also affect the recruitment and activation of immune cells in the tumor microenvironment [143]. SASP can both promote and suppress the immune response and its use as a “double-edged sword” can link cellular senescence to immune response regulation. Given the important role of YBX1 in cellular senescence and immune regulation, understanding how immune cells respond to SASP factors mediated by YBX1 and whether combined modulation of YBX1 levels and SASP metabolic reprogramming can mitigate malignant progression remains a key scientific question requiring further detailed investigation.

While YBX1 exhibits a variety of functions, new biological roles, and mechanisms are continuously being clarified through research. This functional diversity of YBX1 provides a solid theoretical foundation for its potential as a diagnostic and therapeutic target for various diseases. As the functions of YBX1 are further elucidated, treatment strategies based on this protein are likely to be developed progressively, offering new opportunities and possibilities for clinical treatment and contributing to the enhancement of public health.

## Figures and Tables

**Table 2:** YBX1 plays a role in the immune system.

Category	Mode of Influence	Content of Influence	Source
Molecular regulation of immunity and autoimmune diseases	Regulation of immune molecule expression	Regulation of CCL5, IL-6, IL-2, IL-10, TGFβ, Cxr1 Expression	[94,95,96,97,98,99,100,101,102,103,139,140]
	Autoimmune diseases	SLE	[103,105]
		Type I Diabetes	[104]
Tumor immunity and Immunotherapy	Construction of Tumor Risk Models	HCC	[121,126,127,128,129,130,131]
		PC	[132]
		KIRP	[133]
		PapRCC	[134]
	Anti-tumor immune response and immunotherapy responsiveness	Release of Tumor-associated Antigens	[114,141]
		Regulation of Inflammatory Factors and Immune Cell Activation	[111,112,113,119]
		As a Tumor-associated Antigen	[114,116,117,118]
		Risk Model for Accessing Treatment Responsiveness	[120,121,122,123,124]
		Promoting MDR1 Transcription and P-gp Expression	[125]
	Tumor Immune evasion	Regulation of PD-L1 Expression	[70,109,142]
		Modulation of Immunosuppressive Molecules and Activation of Immune Cells	[110,142]
	Cancer Therapy	Targeted inhibitors	[32,135,136,137]
		STEMVAC trial	[138]

## Data Availability

The data that support the findings of this study are available within the paper.
